# Intestinal Spirochetes Associated With Asymptomatic COVID-19 Infection

**DOI:** 10.7759/cureus.27246

**Published:** 2022-07-25

**Authors:** Olivier Van Houtte, Gabrielle Perrotti, Lindsey Gade, Amanda S Ayers, Robert Lewis

**Affiliations:** 1 Colorectal Surgery, Cheshire Medical Center, Keene, USA; 2 General Surgery, Jefferson-Abington Memorial Hospital, Abington, USA; 3 Colorectal Surgery, St. Francis Hospital - Trinity Health of New England, Hartford, USA; 4 Colorectal Surgery, St. Francis Hospital and Medical Center, Hartford, USA

**Keywords:** covid-19, infectious diarrhea, opportunist infections in hiv, human intestinal spirochetosis, infectious colitis

## Abstract

A 60-year-old, human immunodeficiency virus (HIV)-negative, homosexual male presented to our colorectal clinic with abdominal pain for three weeks followed by persistent watery diarrhea refractory to loperamide. He had no history of recent travel, no known infectious contacts, and his last colonoscopy nine years prior was within normal limits. After one episode of hematochezia, computed tomography of the abdomen/pelvis was performed demonstrating colitis and coronavirus disease 2019 (COVID-19)-related changes to the lung bases. Testing confirmed COVID-19 infection which was self-limited. The initial workup for infectious colitis was negative. Colonoscopy revealed no evidence of gross colitis. Histopathology demonstrated microscopic colitis with spirochete colonization of the intestinal epithelium. A course of metronidazole led to the resolution of the patient’s symptoms.

Intestinal spirochetosis has been described as a rare source of colitis caused by the organism *Brachyspira pilosicoli* in an immunocompromised population (HIV-positive, organ transplant). It is associated with abdominal pain and refractory diarrhea. This report details the unique case of intestinal spirochetosis in an HIV-negative, COVID-19-positive patient with no other risk factors for immunosuppression. Further review is necessary to establish a true association; however, this case suggests that intestinal spirochetosis should be considered during the workup of chronic diarrhea (more than two weeks) in COVID-19-positive patients.

## Introduction

Intestinal spirochetosis in humans remains a topic of debate regarding its clinical significance. It has been described in asymptomatic hosts, often found during screening colonoscopies. Whether it exerts a pathological influence is more difficult to ascertain. The majority of infected patients are asymptomatic. However, it has been attributed as the causal agent for symptoms of acute or chronic diarrhea, hematochezia, and abdominal pain. Intestinal spirochetosis is commonly associated with patients who are human immunodeficiency virus (HIV)-positive, engage in men who have sex with men (MSM) sexual behavior, and are immunosuppressed.

More commonly diagnosed in developing countries, intestinal spirochetosis is a rare cause of colitis in the western world. The infection is caused by *Brachyspira pilosicoli*. It is transferred via the fecal-oral route or through ingestion of infected animal products. It can be diagnosed with a tissue biopsy of the terminal ileum or colon or via polymerase chain reaction (PCR) testing. Histology of the tissue biopsies reveals spirochetes attached to the membrane of colonocytes. Patients may choose to be monitored clinically if they are asymptomatic. In symptomatic patients, successful treatment has been reported after a course of metronidazole [1].

## Case presentation

A 60-year-old male presented to the outpatient colorectal surgical clinic with ongoing diarrhea for several weeks and prolonged cramping abdominal pain. His history was significant for unprotected intercourse with men but he had recently tested negative for gonorrhea, chlamydia, HIV, and herpes simplex virus (HSV). He noted no recent travels and had undergone a colonoscopy with no significant findings nine years ago.

Six weeks prior to his outpatient appointment, he had been admitted to the emergency department with abdominal pain. At the time he received an enema which resulted in hematochezia. This prompted the patient to be scheduled for computed tomography (CT) scan of his abdomen and pelvis. Imaging showed that the patient had colitis involving the majority of the colon.

Additionally, the lung bases seen in the abdominal cuts of the CT scan were concerning for coronavirus disease 2019 (COVID-19)-related changes. Further infectious disease testing confirmed that the patient was positive for COVID-19, which was self-limited as he has no respiratory symptoms. He denied any symptoms or history of sick contacts as well. He had not yet been vaccinated against COVID-19.

The patient was discharged from the emergency department with a diagnosis of colitis in addition to COVID-19. He was not prescribed any medications at discharge and did not undergo any further procedures at this time. The patient was instructed to follow up with a colorectal surgeon in six weeks if he remained symptomatic.

For the next six weeks, until his outpatient appointment, he continued to have persistent abdominal pain in his low pelvic and suprapubic abdominal regions. He noted that the pain was aggravated with eating. Additionally, the patient had developed fecal urgency which did not improve with time. Due to his prolonged and worsening symptoms, during his appointment with a colorectal surgeon, he was instructed to undergo further workup. This included stool studies for ova and parasites, *Clostridium difficile*, HSV, gonorrhea, and chlamydia. The workup was negative.

**Figure 1:**
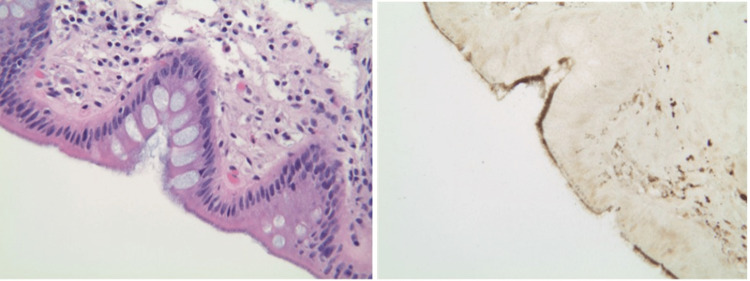
Left: Hemotoxylin and eosin stain demonstrating a blue fringe characteristic of intestinal spirochetosis due to brush border thickening. Right: Warthin-Starry stain demonstrating the “false brush border” associated with intestinal spirochetosis.

He was then scheduled for a colonoscopy. Colonoscopic findings showed normal colonic mucosa with no signs of colitis. Random biopsy samples were taken to rule out a diagnosis of microscopic colitis. Histopathology demonstrated microscopic colitis with spirochete colonization of the intestinal epithelium (Figure 1).

The diagnosis of intestinal spirochetosis was then made and the patient completed a course of metronidazole with full resolution of his symptoms after a 10-day antibiotic course.

## Discussion

Human intestinal spirochetosis, also referred to as colonic spirochetosis, is characterized by anaerobic spirochetes (*B. aalborgi* and *B. pilosicoli*) that colonize the colorectal epithelium. It was first described in the 17th century by van Leeuvenhok in his own diarrheal stools. Harland and Lee then officially coined the term “intestinal spirochetosis” in 1967 [1].

More commonly seen in veterinary medicine, intestinal spirochetosis in humans is more prevalent in developing countries, specifically in India and parts of Asia [2]. Western countries have only noted a 2-7% incidence in colonic biopsy specimens. In the United States, homosexual males have the highest prevalence overall, with a colonization rate of 62.5%. Although researchers have tried to make a connection regarding sexual transmission, none has been identified [3]. Heterosexual adults in multiple countries, including the United States, Italy, and France, have also been noted to contract the disease. Fetal infections have even been documented during pregnancy and were thought to be a sequela of ascending trans-amniotic infection [1].

As previously mentioned, the majority of cases are incidental findings with patients cited as asymptomatic carriers. The most commonly reported symptom is chronic, watery, non-bloody diarrhea. Other reported symptoms can include one or all of the following: constipation, abdominal pain, hematochezia, and alternating between constipation and diarrhea. Watery diarrhea is thought to be a sequela of the spirochetes destroying the colonic microvilli resulting in the loss of absorptive area [3].

Oftentimes, gross anatomical colonoscopy findings are nonspecific. Patients may present with polypoid lesions, erythematous areas, or even normal mucosa. A biopsy that contains the spirochetes is the key to a diagnosis. Hematoxylin and eosin stains of the biopsies are used to visualize the spirochetes under light microscopy. The surface of the colonic epithelium shows blue fringes in a palisade-like arrangement that are characteristic. Electron microscopy shows the spirochetes embedded into the individual colon cells, docking themselves into the cell membrane between destroyed microvilli [3]. Essentially, the villi of the epithelium become blunted from the spirochetes and the mitochondria of cells swell and develop glycocalyx defects. The greater the burden of spirochete attachment, the more cells get destroyed, which correlates with worsening severity of patient symptoms [4]. Recently, PCR has been used to amplify rRNA and detect *B. pilosicoli* from colonic feces. Fluorescent in-situ hybridization with oligonucleotide can target the rRNA of the spirochete species as well [3].

Although treatment in asymptomatic patients is not necessary, patients can undergo clinical monitoring if they do not opt for treatment. In severely symptomatic patients, treatment with metronidazole 500 mg four times a day for 10 days is recommended. This prescription normally accounts for successful treatment and eradication of the spirochetes, as seen in our patient. However, if there is no improvement, a course of macrolides and clindamycin can be considered [3]. There is no need for patients to undergo further monitoring or screening once their symptoms have resolved.

After an extensive literature review, it appears that our patient represents the first reported patient infected simultaneously with COVID-19 and symptomatic spirochetosis. Initially, it was thought that the patient’s diarrhea was a symptom of his COVID-19 infection. Of note, it has been reported in some cases that COVID-19-induced diarrhea can occur and is said to normally last no longer than two weeks. However, because our patient’s diarrhea continued for multiple weeks, his symptoms could no longer be attributed to COVID-19 and further workup was undertaken.

We hypothesize that the patient’s COVID-19 infection led to an immunocompromised state which led to a symptomatic intestinal spirochetosis infection. It is unclear whether the patient was colonized with *B. pilosicoli* which became pathogenic while immunocompromised or the patient contracted *B. pilosicoli* via anal intercourse. As mentioned, our patient had no medical history of being immunocompromised secondary to known medical diseases or medications, nor did he endorse having had anal intercourse. Interestingly, he was asymptomatic from COVID-19, but symptomatic from his spirochetosis infection.

Studies report that the molecular changes that occur in the beginning stages of a COVID-19 infection result in immunosuppression and tight junction impairment. This resolves in the latter stages of the disease when the lungs have been damaged, and after a chemokine storm has amounted and the patient’s immune response becomes fully activated again. Immunoglobulin lambda variable 3-25 and elongation of factor 1-alpha 1 are thought to be two proteins that play a role in the two stages of the disease [5].

From a molecular standpoint, severe acute respiratory syndrome coronavirus 2 acts at multiple levels to counteract the body’s innate immune system. Essentially, the virus carries multiple proteins that work to suppress the primary trigger for the innate and adaptive immune systems - the induction of interferon. This accounts for immune suppression and leads to a weak antibody response in infected patients to COVID-19 itself and potentially other pathogens as well [6]. Because the majority of symptomatic spirochetosis cases in the United States are found in immunocompromised patients, the immunosuppression experienced from our patient’s COVID-19 infection appears to be the only logical explanation for his symptoms.

## Conclusions

Intestinal spirochetosis represents an unusual cause of abdominal pain and diarrhea in humans. It has previously been described in the immunocompromised population and in developing countries. Our patient, an immunocompetent male with concomitant asymptomatic COVID-19 infection, represents a unique case and the first of its kind to our knowledge. The reported symptoms, diagnosis by colonoscopy, and cured with a course of metronidazole reflect the traditional presentation, diagnostic modality, and treatment pathway of intestinal spirochetosis. While diarrhea is a common COVID-19 symptom, persistent diarrhea for more than two weeks should prompt a broader workup that includes ruling out a diagnosis of intestinal spirochetosis.
